# The Book World of Medicine and Science

**Published:** 1895-08-31

**Authors:** 


### THE BOOK WORLD OF MEDICINE AND SCIENCE.

Horne's Guide to Whitby. Profusely illustrated. Price 1s., or paper cover 6d. (Whitby: Horne and Son, 18, Bridge Street.)

Whitby has long been an established favourite among those who look to a sojourn in bracing air to restore their tired faculties. The town itself affords all the amusements proper to seaside resorts and ample accommodation for visitors at reasonable rates, while in many of the surrounding villages very pleasant quarters are to be had of a less conventional nature. Ample information will be found in the above guide on all matters relating both to the town and the outlying district, and the appearance of this fourth edition gives evidence that it supplies a need.

Microbes and Disease Demons. By Edward Berdoe. L.R.C.P.Ed., M.R.C.S.Eng. (London: Swan Sonnenschein and Co., Paternoster Square. 1895.)

Mr. Berdoe brings his heaviest ordnance to bear on the antitoxin treatment. He hits out right, left, and centre, and brings into requisition the services of that stalwart opponent Dr. Lennox Browne to demolish all resistance. The bulk of Mr. Berdoe's book was originally read in the form of a lecture to the members of the Balloon Society during the early part of the year, at a time when there were comparatively few data—derived from clinical experience—on which to base a formidable attack on the method. Of *à priori* arguments, and arguments based on reasoning rather than facts, he brings forward what he seems to think are enough to squash not only the antitoxin treatment, butthe whole gamut of serum-therapeutics. Incidentally Pasteur comes in for undeserved abuse. As the father of serum-therapy he is held responsible for introducing a flagrant system of empyricism, set off with a show of statistices, and backed by the weight of bacteriological institutes. "If," says Mr. Berdoe, "the antitoxin treatment be scientific, I cannot see why the adjective should be denied to Professor Holloway's system, or to Mother Siegel's curative syrup, or Parr's life pills. Perhaps scientific quackery would be an appropriate designation." In Appendix A (written after the delivery of the lecture) a heavy mass of clinical statistics is brought to bear on the antitoxin exponents. In Appendix B the author weakly avails himself of a quotation from the *Star* of June 11th, 1895, and thus brings his argument to a close with a lamentable anti-climax.

A Student's Text-Book of Botany. By S. H. Vines, M.A., D.Sc., F.R.S., Fellow of Magdalen College, and Sherardian Professor of Botany in the University of Oxford. (Swan Sonnenschein and Co. 1895.)

The second part of Dr. Vines' Text-Book of Botany concludes a work which will meet a decided want felt amongst students. It has long been somewhat of a reproach that all our modern botanical text-books deserving of the name have been mere translations of foreign productions, and it is therefore with the greater pleasure that we welcome the volumes issuing from Dr. Vine's pen. Much of the work is most admirable; and especially in the chapter which deals with morphology, we have the best existing account of the present state of our knowledge on this branch of the subject. The part we care least about is the introductory chapter, which treats somewhat formally, perhaps, a topic which really requires discussion. It is hardly necessary to say that the physiological chapters are most excellent. Professor Vines is probably best known as a physiologist, and one can hardly help indulging in a regret that these chapters are not both longer and more numerous. But enough has been said to commend the book to all who wish to find in a small compass a very considerable accumulation of facts concerning plants, their structure and their functions.

